# Hypertrophic scar of the conjunctiva presenting as an eyelid mass: an unusual complication after surgical treatment of a chalazion

**DOI:** 10.1186/s40064-016-3368-x

**Published:** 2016-09-30

**Authors:** Jun Hyuk Son, Joon Hyuk Choi, Su-Ho Lim

**Affiliations:** 1 Department of Ophthalmology, Yeungnam University College of Medicine, Daegu, Republic of Korea; 2 Department of Pathology, Yeungnam University College of Medicine, Daegu, Republic of Korea; 3 Department of Ophthalmology, Daegu Veterans Health Service Medical Center, #60 Wolgok-ro, Dalseo-gu, Daegu, 704-802 Republic of Korea

**Keywords:** Chalazion, Hypertrophic scar, Eyelid mass

## Abstract

**Purpose:**

To present a rare case of a conjunctival hypertrophic scar presenting as eyelid mass after surgical treatment of a chalazion.

**Case presentation:**

A 74-year-old man visited our clinic with severe ocular discomfort and excessive lacrimation since several months in his left eye accompanied by itching of the upper eyelid. Examination of the anterior segment revealed a 6 mm (horizontal) × 8 mm (vertical) round, immobile, hard, mushroom-shaped protruding mass on the tarsal conjunctiva of his left eye. There was no associated pigmentation, ulceration, or tenderness. Excisional biopsy of the benign conjunctival tumor was performed using radiofrequency electrosurgical systems. The region of the excised conjunctiva was well-healed on postoperative day 14, and there was no recurrence until 1 year post-surgery. Histopathological examination demonstrated thick interlacing collagenous fibrous bundles oriented in random directions and fibroblastic proliferation. Immunohistochemical staining revealed that spindle-shaped fibroblasts were positive for CD34 and negative for smooth muscle actin. The excessive collagenous tissue was stained blue by Masson trichrome stain. These findings were consistent with a hypertrophic scar of the conjunctiva.

**Conclusion:**

This short report demonstrates that a hypertrophic scar of the conjunctiva can develop after surgery of a chalazion and cause severe ocular discomfort and excessive lacrimation. These lesions can be easily removed using simple excision.

## Background

Hypertrophic scars of conjunctival tissues following ocular surgery do not develop commonly (Lyu et al. 2013; Urban and Kaufman 1994; D’Hermies et al. 2003). To date, there have been few reports of hypertrophic scars after chalazion surgery (D’Hermies et al. 2003).

## Case presentation

A 74-year-old man visited our clinic with severe ocular discomfort and excessive lacrimation since several months in his left eye accompanied by itching of the upper eyelid. Four months prior, he underwent surgery for a chalazion secondary to posterior blepharitis.

**Fig. 1:**
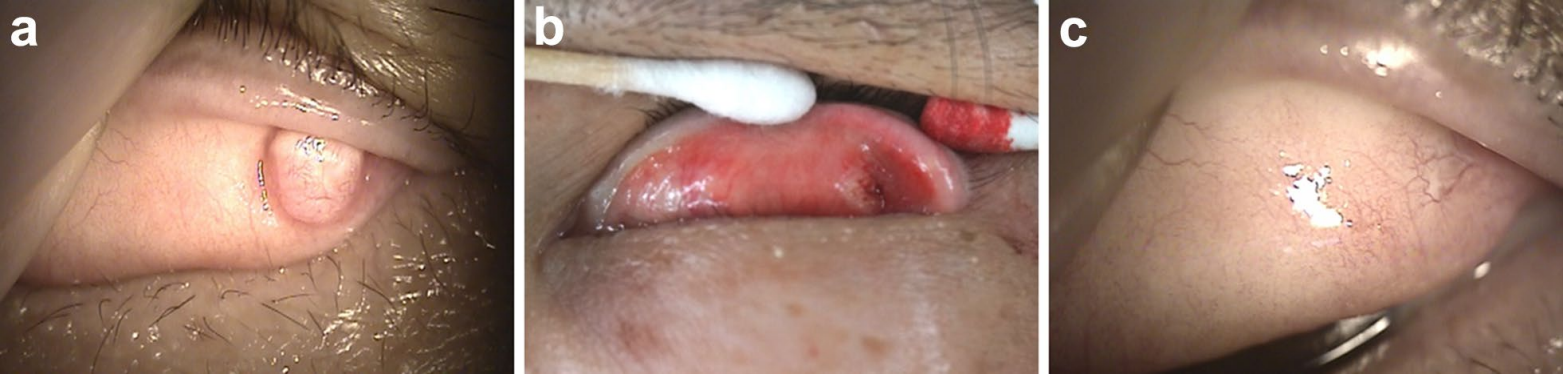
Anterior segment photography of a conjunctival hypertrophic scar at preoperative (**a**), immediate postoperative (**b**), and 14 days postoperative (**c**)

Examination of the anterior segment revealed a 6 mm (horizontal) × 8 mm (vertical) round, immobile, hard, mushroom-shaped protruding mass on the tarsal conjunctiva of his left eye. There was no associated pigmentation, ulceration, or tenderness (Fig. 1a). Excisional biopsy of the benign conjunctival tumor was performed using radiofrequency electrosurgical systems (Ellman International Inc., Oceanside, NY) under local anesthesia (Fig. 1b). The region of the excised conjunctiva was well-healed on postoperative day 14 (Fig. 1c), and there was no recurrence until 1 year post-surgery.

**Fig. 2:**
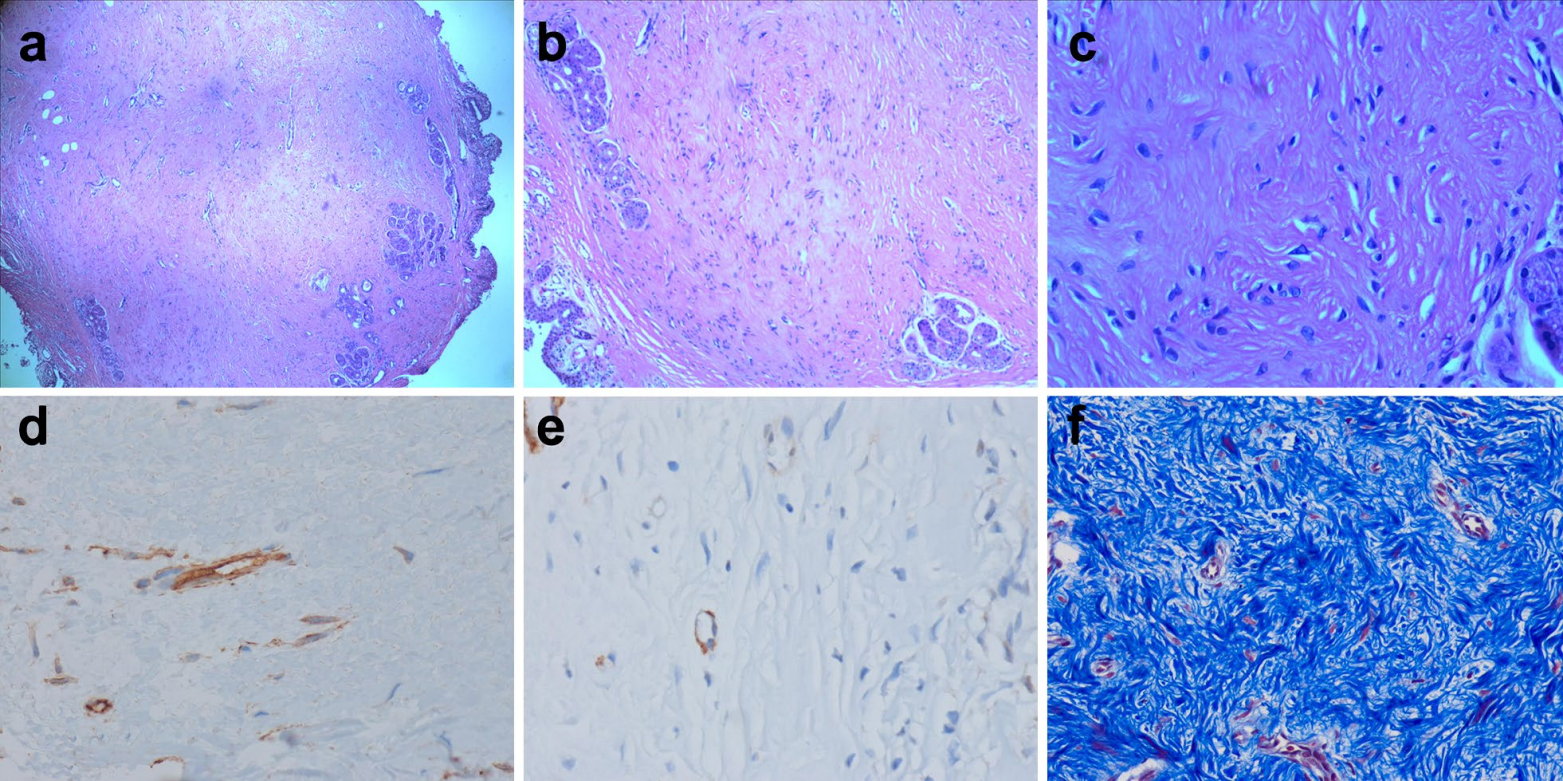
Histopathological examinations (**a**–**c**) demonstrated the diffuse proliferation and infiltration of collagenous fibrous bundles (hematoxylin and eosin, ×40, ×100, ×400). Spindle-shaped fibroblasts are positive for CD34 (**d**; immunohistochemical staining, ×400) and negative for smooth muscle actin (**e**; immunohistochemical staining, ×400). The excessive collagenous tissue is stained *blue* (**f**; Masson’s trichrome staining, ×200)

On microscopic examination, the lesion was composed of spindle-shaped fibroblasts and collagenous stroma (hematoxylin & eosin stain, 40×, Fig. 2a). A section of the specimen showed thick, interlacing, collagenous fibrous bundles oriented in random directions and fibroblastic proliferation (Fig. 2b, c). Immunohistochemical staining revealed that spindle-shaped fibroblasts were positive for CD34 and negative for smooth muscle actin. The excessive collagenous tissue was stained blue by Masson trichrome stain (Fig. 2d–f). These findings were consistent with a hypertrophic scar of conjunctiva. And there was no evidence of the malignancy.

## Discussion

Conjunctival hypertrophic scars following ophthalmic surgery are rare (Lyu et al. 2013; Urban and Kaufman 1994; D’Hermies et al. 2003). In previous studies, cryotherapy for retinopathy of prematurity (Lyu et al. 2013), strabismus surgery including peritomy (Urban and Kaufman 1994), or surgical treatment for chalazion (D’Hermies et al. 2003) were shown to cause scarring of the conjunctiva or Tenon’s capsule. As in our case, D’Hermies et al. (2003) reported a whitish mass that gradually developed on the conjunctival face of the eyelid disturbing the use of contact lens, which was diagnosed as a “hypertrophic eyelid conjunctival scar”. A hard and fibrous lesion was resected under local anesthesia in their report. In our case, we performed a similar simple resection using radio frequency electrosurgery.

Differential diagnosis includes keloid and angiofibroma. Keloid is less cellular and has thicker collagen fibers. Angiofibroma is characterized by small vessel dilatation and concentric perifollicular fibrosis. The histologic features of the present case are compatible with hypertrophic scar.

The eyelids are dynamic and complex structures. The primary function of eyelid is to protect the ocular surface via cleansing and lubrication of eyes. They serve as both a physical and immunological barrier, which constitutes a crucial defense mechanism (Lin et al. 2011). In these contexts, a hypertrophic scar on the tarsal side of the conjunctival tissue presenting as an eyelid mass could cause tear film instability and secondary ocular surface disease.

## Conclusion

This case report demonstrated that a hypertrophic scar of the conjunctiva can develop after surgical treatment for a chalazion and cause severe ocular discomfort and excessive lacrimation. These lesions can be easily removed using simple excision.
